# Supplementary material for: The transcriptional coactivator RUVBL2 regulates Pol II clustering with diverse transcription factors
Source: Nat Commun. 2022 Sep 28;13:5703. doi: 10.1038/s41467-022-33433-3 (PMC9519968; doi:10.1038/s41467-022-33433-3)
Supplement: Supplementary file 1 — Supplementary Information [file 41467_2022_33433_MOESM1_ESM.pdf]

Supplementary Information

Supplementary Fig. 1

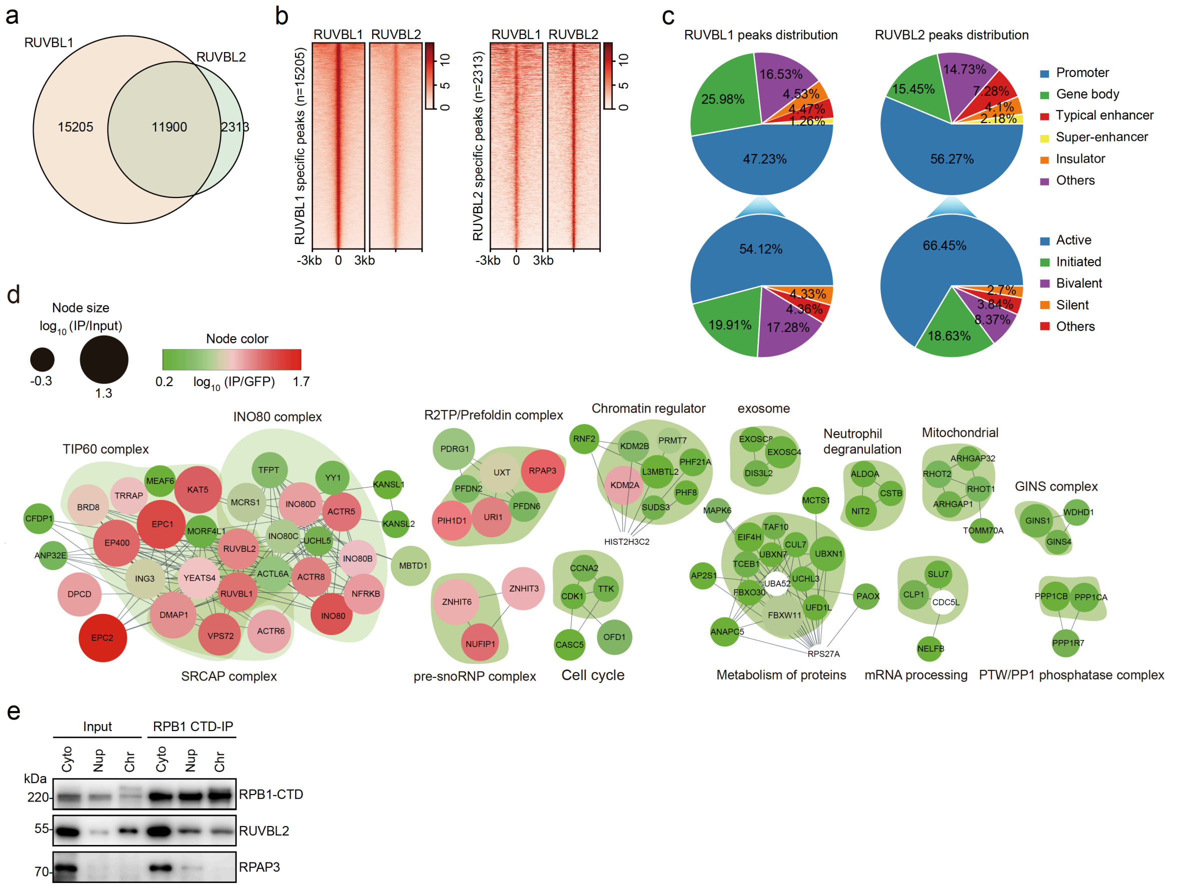

Supplementary Fig. 1 | Genomic and proteomic profiling of RUVBL2 in mESCs.

a. Comparison of RUVBL1 and RUVBL2 ChIP-Seq peaks. Venn diagram illustrating the number of overlapping RUVBL1 and RUVBL2 ChIP-Seq peaks in this study.

b. Heatmaps of RUVBL1 and RUVBL2 ChIP-Seq signals at RUVBL1-specific peaks (left panel) and RUVBL2-specifics (right panel).

c. The distribution of RUVBL1 and RUVBL2 peaks in various regulatory elements (upper panel) and various promoter categories (bottom panel) in mESCs. Detailed definitions of regulatory elements and promoter categories can be found in the Methods section.

d. The protein complexes detected in RUVBL2 ChIP-MS preparations. The node size indicates the $\log_{10}$ value for the ratio of the number of peptides in the input samples to the number of peptides in the RUVBL2 ChIP-MS preparations. The color indicates the $\log_{10}$ value for the ratio of the number of peptides in the RUVBL2 ChIP-MS preparations to the number of peptides in the GFP ChIP-MS control samples. The lines indicate the protein–protein interactions detected in the STRING database. The white nodes indicate proteins that did not satisfy our cutoff criteria or were not detected by RUVBL2 ChIP-MS. The green background for each category indicates the members that had been reported to be the

corresponding complexes or pathways; the placement of a protein outside of the green background indicates that it has not been reported to be in the related complexes but showed close interactions with the corresponding complexes or pathways in the STRING database.

e. Western blotting was performed to examine RUVBL2 and RPAP3 subunits in RPB1 immunoprecipitated from different subcellular fractions. Cyto is the cytoplasm; Nup is the nucleoplasm; and Chr is the chromatin fraction. RPB1 was the control. Data represented 3 times experiment repeated independently with similar results.

Supplementary Fig. 2

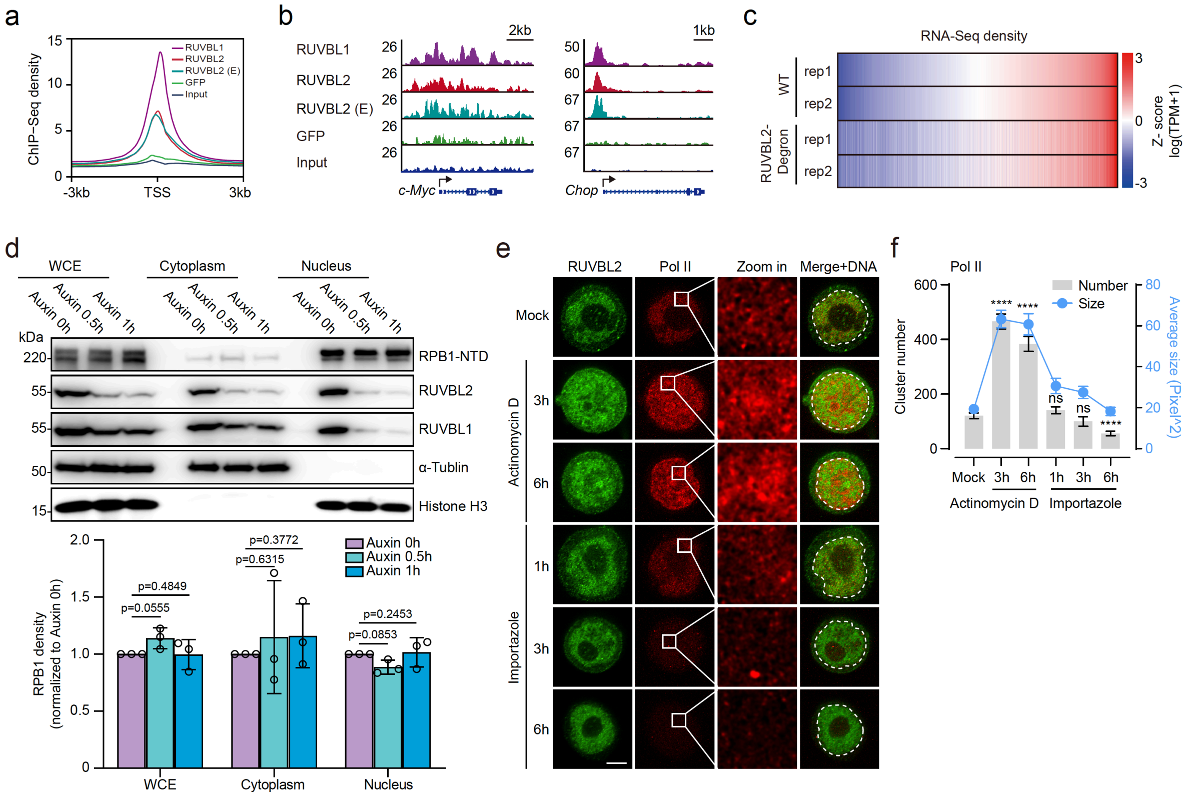

Supplementary Fig. 2 | Marked depletion of RUVBL2 did not affect the nuclear abundance of RPB1.

a. The results of meta-analyses of ChIP-Seq signals of GFP-degron RUVBL1 and GFP-degron RUVBL2 (GFP antibody ChIP), antibody against endogenous RUVBL2 (E), GFP control (GFP antibody used in ChIP-Seq with wild-type mESCs), and input at transcription start sites (TSSs).

b. ChIP-Seq snapshots showing GFP-degron RUVBL1, GFP-degron RUVBL2, endogenous RUVBL2 (E), GFP control, and input at c-Myc and Chop loci in mESCs.

c. The gene expression profile identified by RNA-Seq in RUVBL2-degron cells was very similar to that in wild-type cells. The heatmap was sorted by the RNA-Seq signals in wild-type mESCs.

d. Nuclear and cytoplasmic fractions of Pol II (RPB1) and RUVBL1/2 were examined after marked RUVBL2 depletion. α-Tubulin and histone H3 were used as cytoplasmic and nuclear markers, respectively. The relative Pol II densities in the whole-cell extract (WCE) and nucleus were normalized to the density of histone H3, and the cytoplasmic Pol II densities were normalized to the density of α-Tubulin. Then, the data obtained after each auxin treatment were further normalized to that obtained at 0 h. The statistical significance was determined by two-tailed Student's t test on the basis of replicate experiments (n=3), data are presented as mean +/- SD.

e. Representative images of RPB1 changes after actinomycin D treatment or importin inhibition after importazole treatment. Scale bars, 3 μm.
f. The bar graph shows the number of Pol II clusters and their sizes after different treatments. Statistical significance was determined from at least 10 field cells (Mock n=21; Actinomycin D 3h n=25, 6h n=24; Importazole 1h n=19, 3h n=22, 6h n=23). Significance was analyzed by two-tailed Student's t test (ns, not significant, ****p < 0.0001), error bar indicates the +/- SD, and the exact p values can be found in the source data.

Supplementary Fig. 3

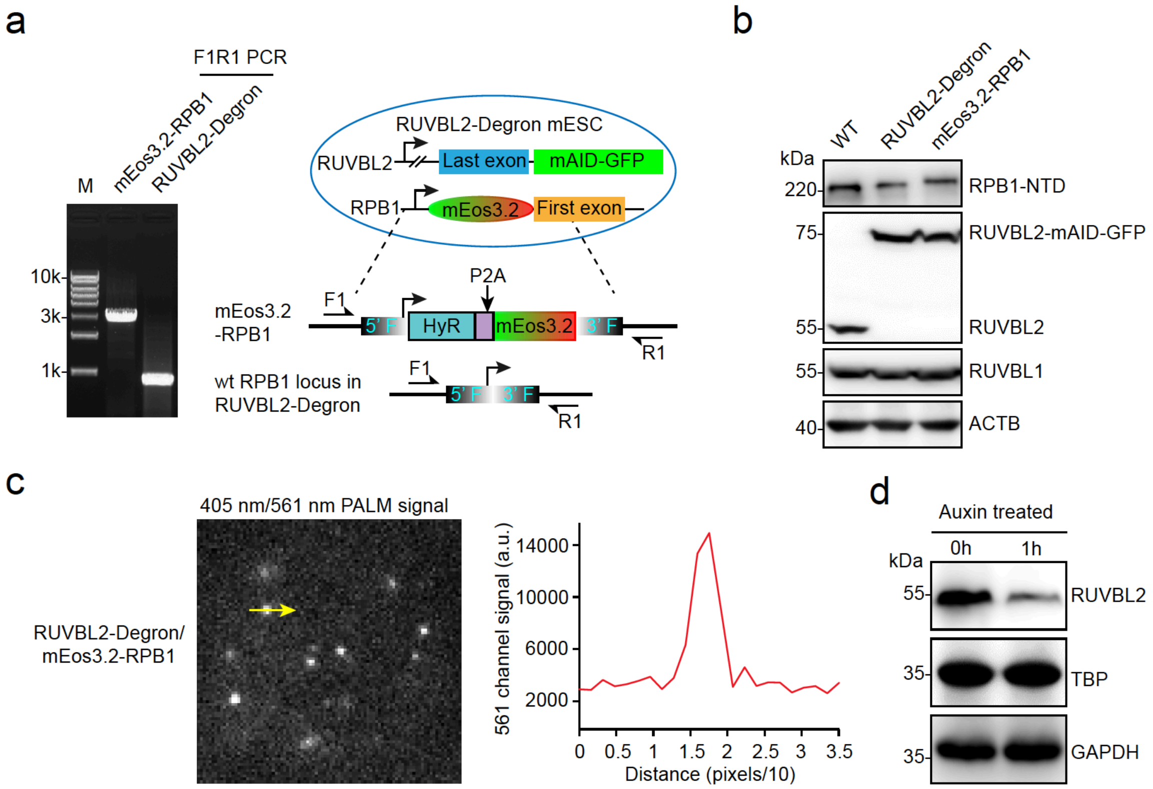

Supplementary Fig. 3 | Characterization of mEos3.2-RPB1 mESCs.

a. An mEos3.2 tag was knocked into the N-terminus of RPB1 in RUVBL2 degron mESC (the graphic diagram), Hygromycin resistance (HyR) and mEos3.2 were divided by P2A tag, the genotyping primers "F1/R1" were located lateral side of 5' and 3' homologous arm (5'F and 3'F), respectively, the F1/R1 PCR products of mEos3.2-RPB1 knocked in are 2674 base pairs closed to the 3k DNA ladder, while if not knocked in, the F1/R1 PCR products are 898 base pairs (wt RPB1 locus) which neared to 1k DNA ladder. The electrophoresis of PCR genotyping assay showed that the mEos3.2-RPB1/RUVBL2 degron was homozygous, the mEos3.2-RPB1 band was extracted and confirmed by Sanger sequencing.
b. Western blotting was performed to measure the protein levels of RPB1, RUVBL1 and RUVBL2 in wild-type, RUVBL2-degron and mEos3.2-RPB1 mES cells. ACTB was used as a loading control.
c. mEos3.2 was photoconverted using a 405 nm-/561-nm laser pair. The raw image showed that activated mEos3.2 had a good signal-to-noise ratio (right), and signals are shown along the yellow arrow in the right panel. The marked peak distribution (Gaussian) of signals suggests that one mEos3.2-RPB1 molecule was detected at this locus at this time point.
d. The TBP protein level was examined with Western blotting after acute RUVBL2 degradation for 1 h, and GAPDH was a loading control. Data represented 2 times experiment repeated independently with similar results.

Supplementary Fig. 4

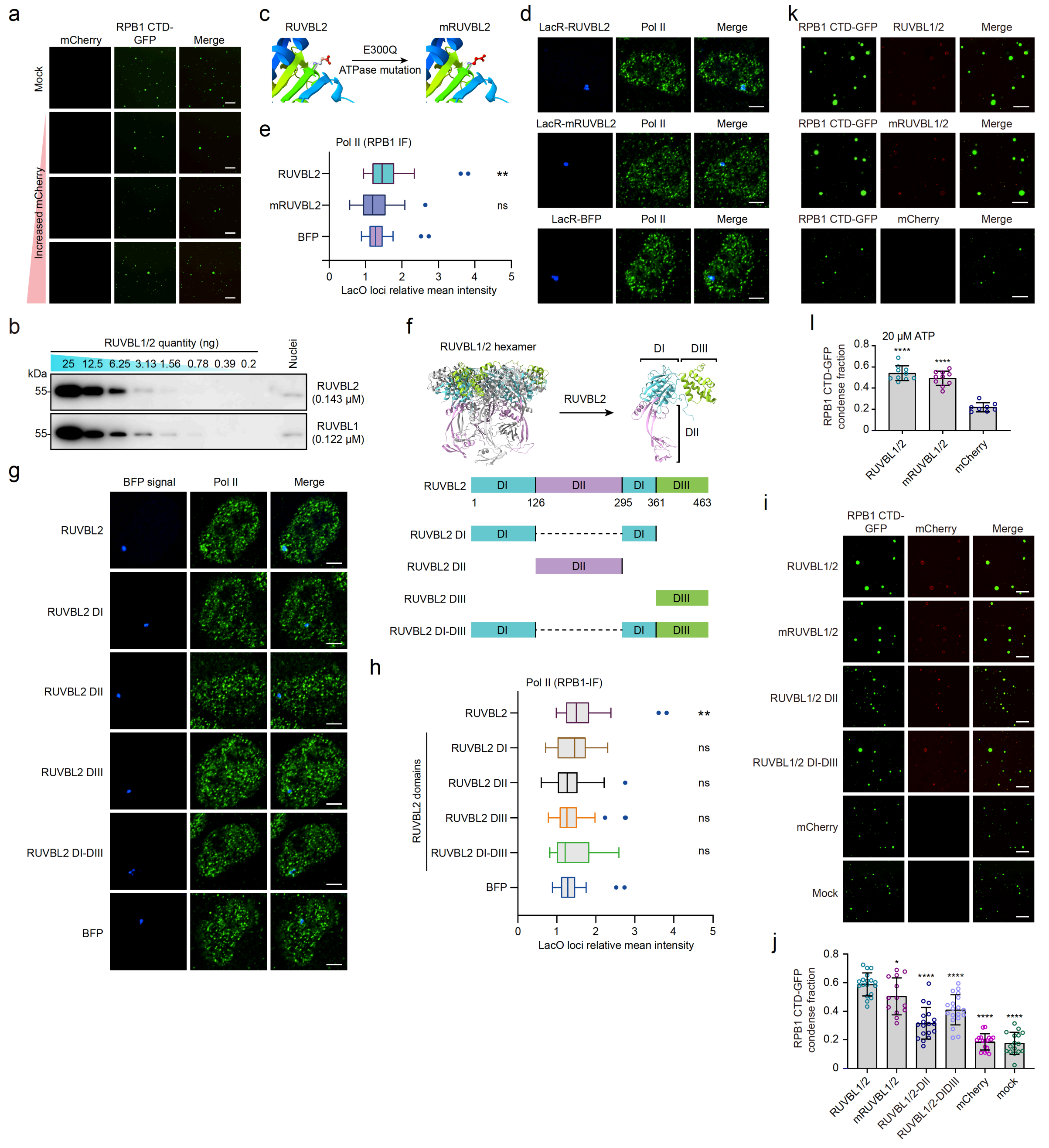

Supplementary Fig. 4 | RUVBL2 domain dissection to investigate the contributions of different domains to Pol II clustering.

a. Representative images of mCherry proteins in the RPB1-CTD droplet assay. The scale bar is 20 µm.

b. The nuclear concentration of RUVBL1/2 was quantified through reference to the recombinant purified RUVBL1/2 complex.

c. The ATPase core amino acid E300 was replaced with a Q residue generate a RUVBL2 ATPase mutant (PDB ID: 6H7X).

d. Representative images showing the tethering experiments with the RUVBL2 ATPase mutant. The scale bar is 5 µm.

e. Relative enrichment of Pol II at the LacO loci. More than 10 different fields cells were assessed for the calculation and statistical analyses (RUVBL2 n=37, mRUVBL2 n=43, BFP n=36). Two-tailed Student's t test was carried out to determine the significance, **p<0.01; “ns” indicates no significant difference, the exact p value can be found in the source data.
f. Diagram of the RUVBL2 domains. DI, DII, and DIII indicate domains I, II and III, respectively (PDB ID: 6IGM).
g. Representative images showing tethering experiments with different RUVBL2 domains. The scale bar is 5 μm.
h. Relative enrichment of Pol II at the LacO loci after tethering of different RUVBL2 domains. More than 10 different fields cells were used for the calculation and statistical analyses (RUVBL2 n=37, RUVBL2 DI n=41, RUVBL2 DII n=55, RUVBL2 DIII n=36, RUVBL2 DIDIII n=31, BFP n=36). Two-tailed Student's t tests were carried out to compare BFP and other conditions. **p<0.01, *p<0.05, “ns” indicates not significantly different.
i. Representative images of different RUVBL2 domains in the RPB1-CTD droplet assay. The scale bar is 20 μm.
j. The condensed fractions of RPB1 CTD signals under the conditions displayed in Supplementary Fig. 4i. The condense fraction was calculated from different fields (RUVBL1/2 n=18, mRUVBL1/2 n=13, RUVBL2 DII n=18, RUVBL2 DIDIII n=19, mCherry n=17, Mock n=16) and statistically analyzed using two-tailed Student's t test. *p< 0.05, **p< 0.01, ***p< 0.001, ****p<0.0001, data are represented as mean +/- SD.
k. Representative images showing the ATPase-mutant RUVBL1/2 proteins in the RPB1-CTD droplet assay. The scale bar is 20 μm.
l. The condensed fractions of RPB1 CTD signals in Supplementary Fig. 4k. Statistical analysis was based on the different fields (RUVBL1/2 n=10, mRUVBL1/2 n=10, mCherry n=8) and is shown as points in histogram. Two-tailed Student's t tests were used. ****p<0.0001, data are represented as mean +/- SD.

Supplementary Fig. 5

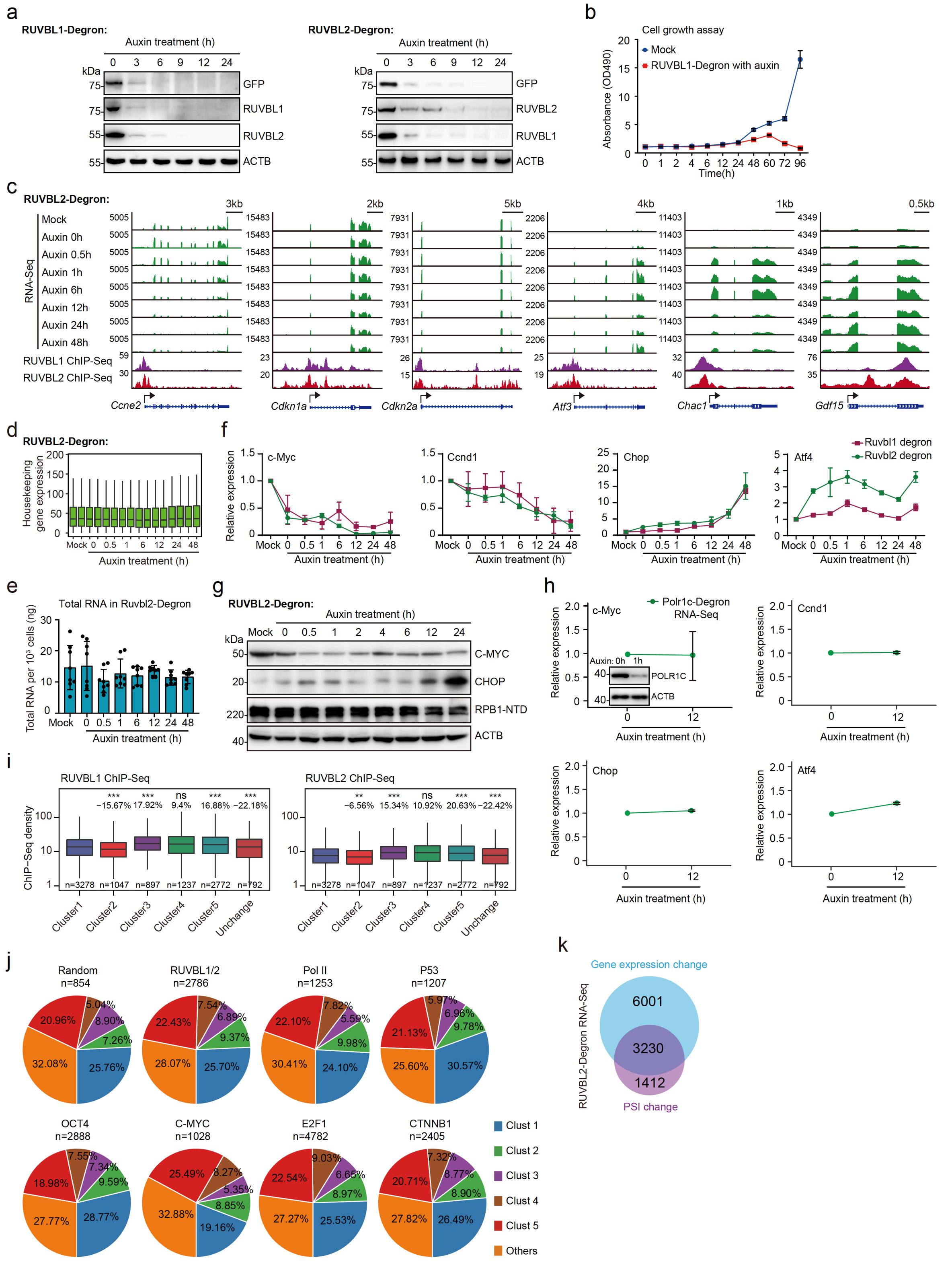

Supplementary Fig. 5 | RUVBL2 depletion leads to both up- and downregulation of mRNA expression in mESCs.

a. Western blot analyses showing RUVBL1 and RUVBL2 degradation.

b. Cell growth analyses were performed after RUVBL1 depletion. 3 replicate wells (n=3) per independent experiments (n=3 biological replicates), error bar indicate the +/- SD.
c. Snapshots showing RNA-Seq signals at the Ccne2, Cdkn1a, Cdkn2a, Atf3, Chac1 and Gdf15 loci.
d. The gene expression levels of housekeeping genes (Hounkpe et al., 2019) (n=2928) are displayed as box plots.
e. The amount of total RNAs per 1000 cells was measured 2-3 times at different time points after RUVBL2 degradation. Independent biological replicates (n=3) were used for quantification. The error bars indicate the mean +/- SDs, One-way ANOVA test indicated no significant different among the different time points (p=0.2306).
f. RT–qPCR analyses of c-Myc, Ccnd1, Chop and Atf4 gene expression at different time points after degradation of RUVBL1 (red) and RUVBL2 (green). Independent biological replicates (n=3) were used for quantification. The error bars indicate the mean +/- SDs.
g. Western blot analyses of C-MYC, CHOP and Pol II (RPB1) protein levels during RUVBL2 degradation.
h. Gene expression analyses of RNA-Seq data (n=2) for c-Myc, Ccnd1, Chop, and Atf4 with Polr1c depletion in mESCs, and error bars indicate the mean +/- SEs. Western blot analysis confirmed the degradation of Polr1c in mESCs, as shown in the panel.
i. The RUVBL1/2 ChIP-Seq signals (in Fig. 6f) in the regions ± 200 bp of the TSSs were calculated and plotted as box plots. Two-tailed Wilcoxon test was performed to calculate significance (*p < 0.05, **p < 0.01, ***p < 0.001). The numbers below the asterisk indicate the fold change compared with cluster 1.
j. The numbers of genes in each cluster shown in Fig. 6e that were simultaneously affected by UPF1 knockdown (Hurt et al., 2013) and bound by each corresponding transcription factor were plotted in pie graphs.
k. Venn diagram illustrating the overlapping genes whose expression changed and whose splicing changed after RUVBL2 depletion.

Supplementary Fig. 6

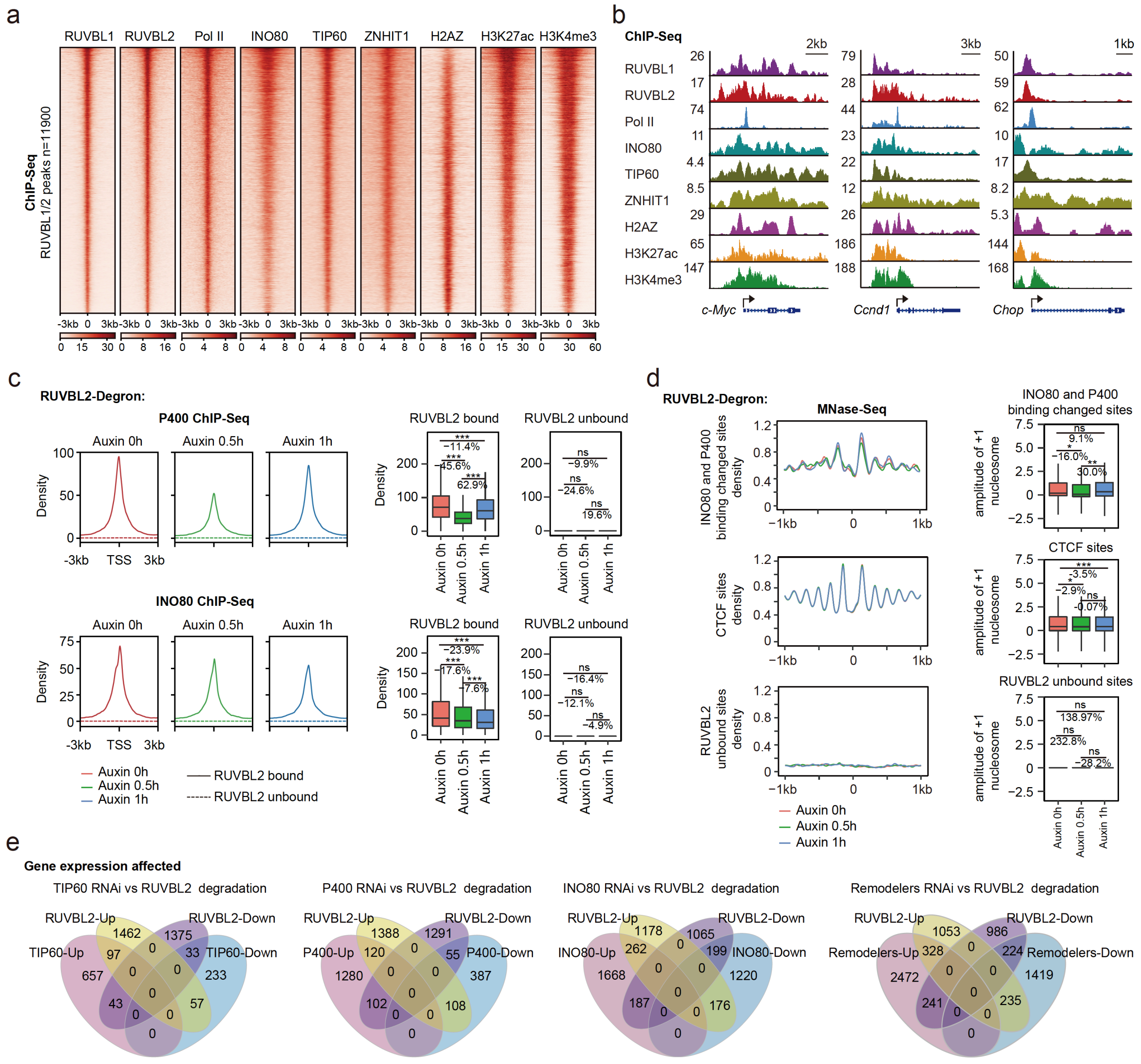

Supplementary Fig. 6 | INO80 and TIP60 complex activities could not directly explain the transcription inhibition observed immediately after depletion of RUVBL2.

a. Heatmap illustrating that RUVBL1 and RUVBL2 globally colocalized with Pol II and INO80/SWR remodelers in mESCs.

b. Snapshots showing ChIP-Seq signals of RUVBL1, RUVBL2, Pol II, INO80, TIP60, ZNHIT1, H2AZ, H3K27ac, and H3K4me3 at the c-Myc, Ccnd1, and Chop loci in mESCs.

c. ChIP-Seq profiles of INO80 (top panel) and P400 (bottom panel) after RUVBL2 degradation at the TSS regions of RUVBL-bound (solid line) (n=1000) and RUVBL-unbound (dashed line) (n=1000) genes. Two-tailed Wilcoxon test was performed to calculate the significance of differences (ns, not significant, *$p < 0.05$, **$p < 0.01$, ***$p < 0.001$). The center line of boxplot represents median, the box limits represent upper and lower quartiles, and the whiskers represents 1.5x interquartile range or maximum/minimum value.

d. MNase-Seq profiles at INO80 and P400 binding changed sites (top panel) (n=814), CTCF sites (middle panel) (n=54772) and RUVBL2-unbound sites (bottom panel) (n=1000) after RUVBL2 degradation. The MNase-Seq amplitudes for the plus 1 nucleosome region were calculated and plotted as box plots. Two tailed Wilcoxon test was used to calculate the significance of differences (ns, not significant, *p < 0.05, **p < 0.01, ***p < 0.001).
e. Analysis of the overlap of differentially expressed genes between RUVBL2 depletion datasets (auxin for 12 h) and the publicly available TIP60 (Fazzio et al., 2008) (left), P400 (Fazzio et al., 2008) (middle) and INO80 (Wang et al., 2014) (right) knockdown gene expression datasets. The combination of the knockdown remodeler gene sets was also compared with the RUVBL2-degradation gene set (right most).

Supplementary Fig. 7

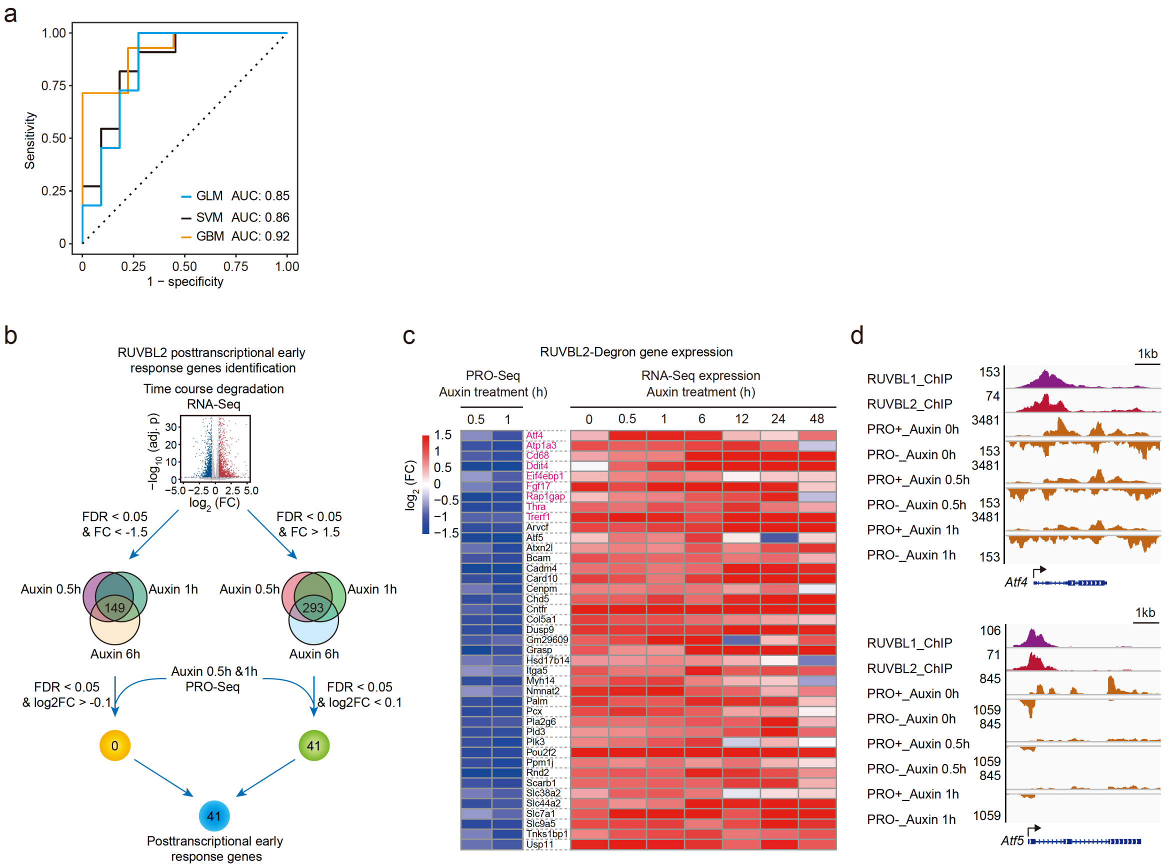

Supplementary Fig. 7 | Identification of the posttranscriptional early response genes regulated by RUVBL2.

a. Receiver operating characteristic (ROC) curves for determining the classification of genes directly targeted by RUVBL2 and nontarget genes based on the test sets for the elastic net generalized linear model (GLM), gradient boosting machine (GBM), and support vector machines with linear regression (SVM) models.

b. The workflow of the process used to identify the posttranscriptional early response genes. To identify genes that were not directly regulated by RUVBL2 but responded very early at the RNA-Seq level, which were the genes with the same RNA-Seq patterns at 0.5 h, 1 h and 6 h but the opposite PRO-Seq patterns, were selected. For posttranscriptional early response downregulated genes (left panel), the downregulated genes identified by RNA-Seq at 0.5 h, 1 h and 6 h (FDR<0.05 & Fold Change (FC) <-1.5) were selected first, and then, the genes that were not decreased in the PRO-Seq analysis (FDR<0.05 & log_2 (FC) >-0.1) were selected. For posttranscriptional early response upregulated genes (right panel), the upregulated genes identified by RNA-Seq at 0.5 h, 1 h and 6 h (FDR<0.05 & FC>1.5) were selected first, and then, the genes that were not increased in the PRO-Seq data (FDR<0.05 & log_2 (FC) <0.1) were selected. Ultimately, 41 posttranscriptional early response genes were identified. The FDR are extracted from DESeq2 by adjusting p values using BH method.

c. Heatmap analyses of posttranscriptional early response genes according to the PRO-Seq expression signals at different time points after RUVBL2 degradation are shown on the left. The gene names are shown in the middle. The genes associated with the biological process term “cellular response to endogenous stimulus” are labeled in red. The right heatmap shows the RNA-Seq expression signal changes after RUVBL2 degradation at different time points. The heatmap color indicates the normalized signals under each condition compared with the auxin 0 h condition.
d. Snapshots showing the RUVBL1 and RUVBL2 ChIP-Seq signals and nascent RNA (PRO-Seq) signals after 0.5 h and 1 h of RUVBL2 degradation at the Atf4 and Atf5 loci.

Supplementary Fig. 8

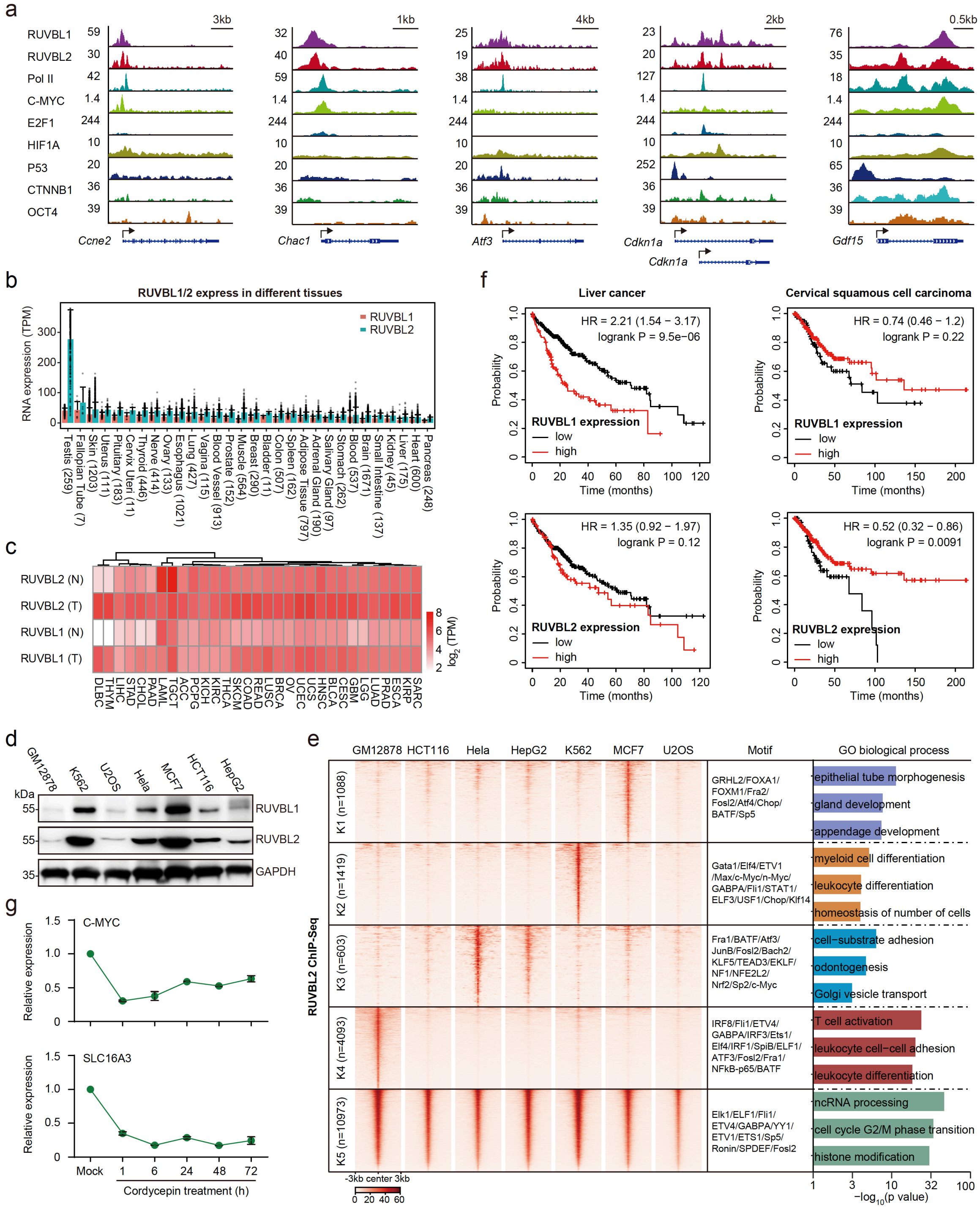

Supplementary Fig. 8 | RUVBL2 exhibits promiscuous co-occupancy with multiple transcription factors and is overexpressed in cancers.

a. ChIP-Seq snapshots showing RUVBL1, RUVBL2, Pol II, C-MYC, E2F1, P53, CTNNB1, and OCT4 at the Ccne2, Chac1, Atf3, Cdkn1a and Gdf15 gene loci in mESCs.

b. The mRNA expression datasets from the GTEx database were used to plot RUVBL1/2 expression in various human tissues. The error bars indicate the SDs

and the expression levels of each sample of each tumor type are marked on the graph as points. And the sample numbers in each tumor type were labeled in the bottom.

c. The mRNA expression levels of RUVBL1 and RUVBL2 in various normal (N) and tumor (T) tissues obtained from the GEPIA database.
d. Western blot analyses of RUVBL1 and RUVBL2 protein levels in different cell lines. Equal numbers of cells were lysed, and GAPDH was used as a loading control.
e. Cluster analyses of RUVBL2 ChIP-Seq signals in various cell lines. The number of genes in each cluster is shown on the left. The enriched transcription factor motifs at the gene transcription start sites of each cluster are shown on the middle; a p value of <0.01 was the cutoff criterion. The enriched GO terms for each cluster are shown in the right; FDR<0.01 was the cutoff criterion. The FDR are extracted from clusterProfile by adjusting p values using BH method.
f. The survival probability changes over time for patients with high (red line) and low (black line) expression of RUVBL1 (upper panel) and RUVBL2 (lower panel) in liver cancer (left panel) and cervical squamous cell carcinoma (right panel) as determined with the Kaplan–Meier plotter. HR indicates the hazard ratio (ratio of the risk of death before and after the expression of RUVBL1/2 was increased by one unit), and the confidence interval is shown in brackets.
g. RT–qPCR analysis of C-MYC and SLC16A3 gene expression in MCF-7 cells. Statistical data were quantified on the basis of 3 biological replicates. The error bars indicate the mean +/- SDs.
