# Supplementary material for: The transcriptional coactivator RUVBL2 regulates Pol II clustering with diverse transcription factors
Source: Nat Commun. 2022 Sep 28;13:5703. doi: 10.1038/s41467-022-33433-3 (PMC9519968; doi:10.1038/s41467-022-33433-3)
Supplement: Supplementary file 3 — Description of additional Supplementary File [file 41467_2022_33433_MOESM3_ESM.pdf]

Descriptions of Additional Supplementary Data files

Supplementary Data 1: RUVBL2 and RPB1 ChIP-MS data summary

Supplementary Data 2: Quality matrix for sequencing data in this study

Supplementary Data 3: RNA-Seq expression summary

Supplementary Data 4: RUVBL1 and RUVBL2 ChIP-Seq peaks in this study

Supplementary Data 5: Public data used in this study

Supplementary Data 6: Oligos used in this study
